# Ethyl Gallate Inhibits Bovine Viral Diarrhea Virus by Promoting IFITM3 Expression, Lysosomal Acidification and Protease Activity

**DOI:** 10.3390/ijms24108637

**Published:** 2023-05-12

**Authors:** Linlin Zhang, Guanghui Yang, Jun Wang, Jialu Zhang, Keyuan Chen, Xiaoran Xiong, Yaohong Zhu, Chuang Xu, Jiufeng Wang

**Affiliations:** College of Veterinary Medicine, China Agricultural University, No. 2 Yuanmingyuan West Road, Beijing 100193, China; zhanglinlin0902@163.com (L.Z.); ygh564701721@163.com (G.Y.); junw123126@163.com (J.W.); jialu_zhang1@hotmail.com (J.Z.); c18336733159@163.com (K.C.); x13811372782@gmail.com (X.X.); zhu_yaohong@hotmail.com (Y.Z.)

**Keywords:** BVDV, ethyl gallate, antiviral, IFITM3, lysosomal acidification, autophagy

## Abstract

Bovine viral diarrhea virus (BVDV) is a highly contagious viral disease which causes economic losses to the cattle industry. Ethyl gallate (EG) is a phenolic acid derivative which has various potentials to modulate the host response to pathogens, such as via antioxidant activity, antibacterial activity, inhibition of the production of cell adhesion factors, and so on. This study aimed to evaluate if EG influences BVDV infection in Madin-Darby Bovine Kidney (MDBK) cells, and to understand the antiviral mechanism. Data indicated that EG effectively inhibited BVDV infection by co-treatment and post-treatment in MDBK cells with noncytotoxic doses. In addition, EG suppressed BVDV infection at an early stage of the viral life cycle by blocking entry and replication steps but not viral attachment and release. Moreover, EG strongly inhibited BVDV infection by promoting interferon-induced transmembrane protein 3 (IFITM3) expression, which localized to the cytoplasm. The protein level of cathepsin B was significantly reduced by BVDV infection, whereas with treatment with EG, it was significantly enhanced. The fluorescence intensities of acridine orange (AO) staining were significantly decreased in BVDV-infected cells but increased in EG-treated cells. Finally, Western blot and immunofluorescence analyses demonstrated that EG treatment significantly enhanced the protein levels of autophagy markers LC3 and p62. Chloroquine (CQ) significantly increased IFITM3 expression, and Rapamycin significantly decreased it. Thus, EG may regulate IFITM3 expression through autophagy. Our results showed that EG could have a solid antiviral activity on BVDV replication in MDBK cells via increased IFITM3 expression, lysosomal acidification, protease activity, and regulated autophagy. EG might have value for further development as an antiviral agent.

## 1. Introduction

Bovine viral diarrhea virus (BVDV), a single-stranded RNA virus of genus Pestivirus within the Flaviviridae family, causes bovine viral diarrhea-mucosal disease (BVD-MD), which has clinically manifested as mucosal necrosis, systemic hemorrhagic illness, diarrhea, persistent infection, and reproductive diseases [1]. Presently, BVDV strains are separated into two types, cytopathic (CP) and non-cytopathic (NCP), according to whether they cause cytopathic lesions or not, of the virus in cell culture [2]. NCP-BVDV strains are more frequently observed in nature and are usually isolated from cattle farms that show clinical manifestations of acute infection [3]. If cows are infected with NCP-BVDV in the first trimester of pregnancy, it will result in a large proportion of persistently infected (PI) cow births [4]. PI cattle are immune-tolerant to the strain of the virus with which they are infected, and they continue to excrete huge quantities of BVDV over their lifetime as a primary provider of virus for BVDV-free feedlots, causing economic losses to the farm [5]. Antiviral drug treatment is an effective strategy to prevent BVDV infection. Therefore, it’s critical to investigate the pathogenic mechanism of BVDV and antiviral drugs for preventing BVDV infection.

The first defense against viral invasion is the innate immune response. Interferons (IFNs) are essential for host immunity against viral infection by promoting the expression of IFN-stimulated genes (ISGs). Interferon-induced transmembrane proteins (IFITMs), a group of small proteins ranging from 14–18 kDa in size, were initially discovered 20 years ago as a by-product of IFNs stimulation [6,7]. In mammalian cells, IFITM3 is found on the membranes of early endosomes, late endosomes, and lysosomes [8]. Subsequent studies have shown that IFITMs are known to have antiviral activity against a multitude of flaviviruses, including hepatitis C virus (HCV), Zika virus (ZIKV), classical swine fever virus (CSFV), and dengue virus (DENV) [9,10,11,12]. Moreover, IFITM3 exhibits antiviral activity against viruses which enter cells via the endosomal pathway. The protein of IFITM3 primarily inhibits cellular invasion of enveloped RNA viruses and prevents viral hemifusion with host membranes at the early stages of virus replication. Meanwhile, the latest reports have shown that proteins of IFITM1/2/3 inhibit CSFV infection, which may be associated with lysosomes [9]. Studies have demonstrated that IFITM3 could bind vesicles that carried incoming viral particles and facilitate late endocytic cargo transport to lysosomes for degradation, thereby preventing viral entry into host cells [13].

Autophagy, an indispensable homeostatic process, is important for the maintenance of host health. However, some viruses can use the autophagosome to complete their own replication. The double membrane structure of the autophagosome provides the physical environment for viral replication, while the virus uses the metabolites and energy produced by autophagy to complete replication [14]. For example, HCV and CSFV can induce autophagosome formation to facilitate viral replication [15,16]. Therefore, autophagy may be a target for drug anti-BVDV replication.

Ethyl gallate, a phenolic acid derivative, is isolated from the natural plant. Polyphenol gallates are secondary metabolites of plant origin, consisting of gallic acid esters. Multiple biological activities of EG have been reported in previous studies, including antioxidant activity, antibacterial capacity, inhibition of the production of cell adhesion factors, and induction of apoptosis in cancer cells [17,18,19]. In addition, other gallate derivatives with antiviral activity have been reported, such as epigallocatechin-3-gallate (EGCG) with potent inhibition against hepatitis virus, porcine circovirus type 2, porcine reproductive and respiratory syndrome virus, porcine epidemic diarrhea virus, and influenza virus [20]. But there is little research on the antiviral activity of EG.

Based on the biological activity of polyphenols, we assessed the contribution of EG to anti-BVDV activity in vitro to identify potential antiviral targets of EG and its potential action mechanisms. The purpose of this research was to explore the anti-BVDV effect of EG, which was found to work better at a concentration of 50 µM. We demonstrated that the antiviral features of EG act on BVDV infection at early stages of the viral life cycle. Furthermore, EG could increase the IFITM3 expression, promote lysosomal protease activity and lysosome acidification, leading to the degradation of the virus, which was associated with mechanisms for fighting the virus.

## 2. Results

### 2.1. EG Inhibits BVDV Infection in MDBK Cells

**Figure 1:**
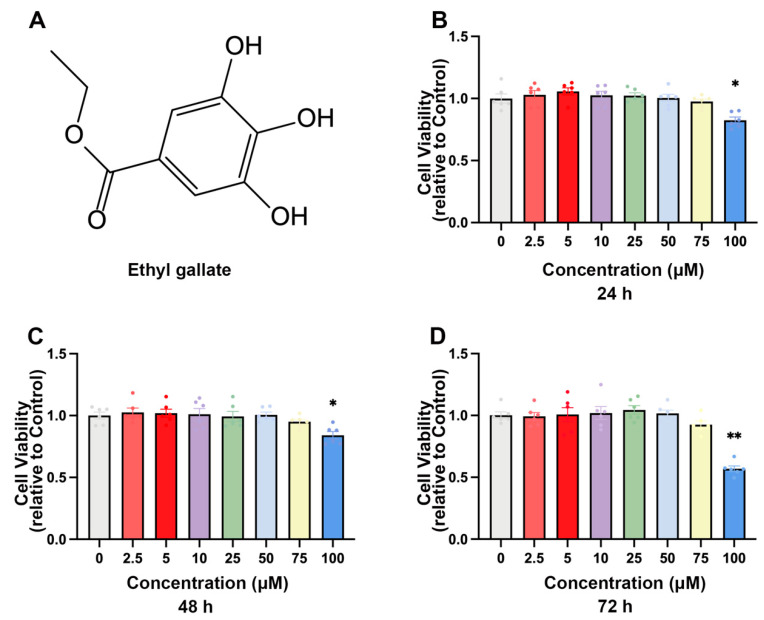
The cytotoxicity of EG on MDBK cells. (**A**) Chemical structure of ethyl gallate. (**B**–**D**) Cell viability was evaluated by CCK-8 assay. MDBK cells were treated with EG 0–100 µM for 24, 48, and 72 h. Data represent mean ± SEM (*n* = 6). * *p* < 0.05, ** *p* < 0.01.

**Figure 2:**
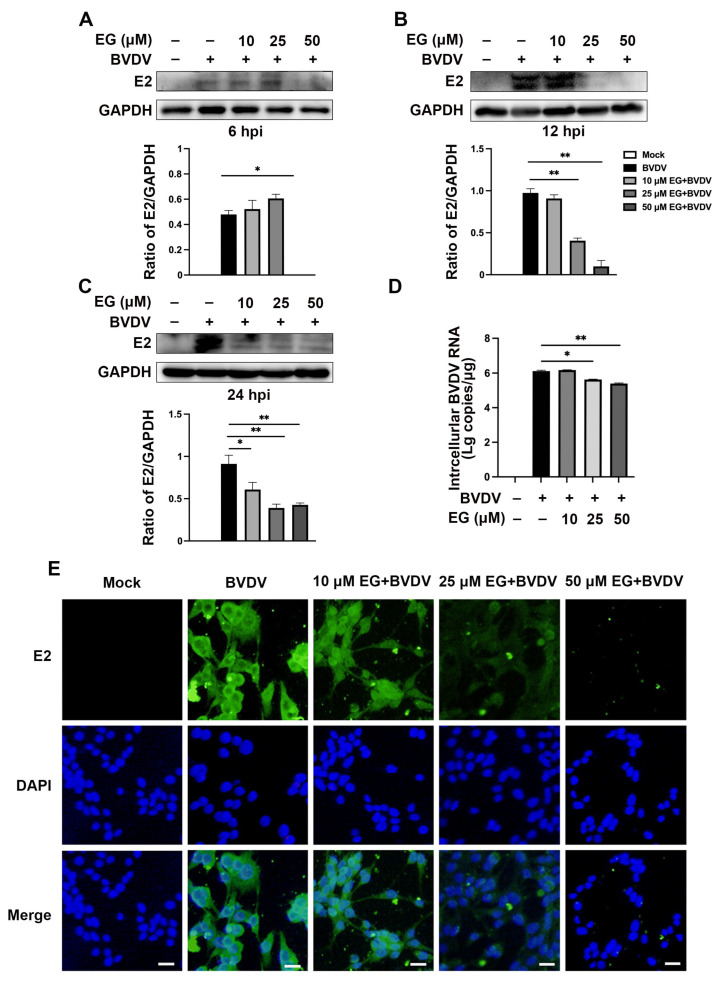
EG inhibits BVDV infection in MDBK cells. (**A**–**C**) Cells were infected with BVDV BJ175170 isolate strains (MOI = 1) and treated with different concentrations (0, 10, 25, 50 µM) of EG for 1 h. At 6, 12, 24 hpi, samples were collected and detected by Western blot. (**D**) The BVDV 5′UTR mRNA level was analyzed by qRT-PCR. Cells were treated with different concentrations (0, 10, 25, 50 µM) of EG for 24 hpi. (**E**) Cells were treated with different concentrations (0, 10, 25, 50 µM) of EG for 24 hpi. Viral replication was analyzed by Immunofluorescence staining. Scale bar, 50 µm. Data represent mean ± SEM (*n* = 3). * *p* < 0.05, ** *p* < 0.01.

The chemical structures of EG, a polyphenolic compound, were used in this study to investigate their inhibitory effect on BVDV infection (Figure 1A). We first evaluated the cytotoxicity of EG (2.5, 5, 10, 25, 50, 75, 100 µM) for 24, 48, and 72 h on MDBK cells by the Cell Counting Kit 8 (CCK-8) assay, respectively. The data showed that the viability of MDBK cells did not significantly decrease at concentrations of 0 to 75 µM (*p* < 0.05) (Figure 1B–D). Therefore, 10, 25, and 50 µM EG were used in subsequent experiments. We then assessed the antiviral activity of EG against BVDV infection using Western blot, qRT-PCR, and Immunofluorescence assays. MDBK cells were exposed to various concentrations of EG (10, 25, and 50 µM) and BVDV (MOI = 1) at different times (6, 12, and 24 h). The Western blot analysis showed that EG effectively reduced the expression level of BVDV E2 protein at a concentration of 50 µM for 6, 12, and 24 h (Figure 2A–C). At 24 h post-infection, the level of intracellular BVDV 5′UTR RNA was analyzed by qRT-PCR. As expected, EG treatment significantly reduced the level of BVDV 5′UTR mRNA expression in a dose-dependent manner (Figure 2D). To obtain a more accurate dose-response curve for EG, MDBK cells were treated with BVDV and EG for 24 h at 37 °C, and fluorescent signals were acquired. As expected, the E2 protein fluorescence signal showed as significantly diminished in the EG-treated group, especially at concentrations of 25 and 50 µM EG, indicating that EG could significantly block BVDV infection (Figure 2E). The above results showed that EG treatment significantly inhibited BVDV infection in vitro at a concentration of 50 µM.

### 2.2. EG Inhibits BVDV Entry and Replication of Viral Life Cycle

**Figure 3:**
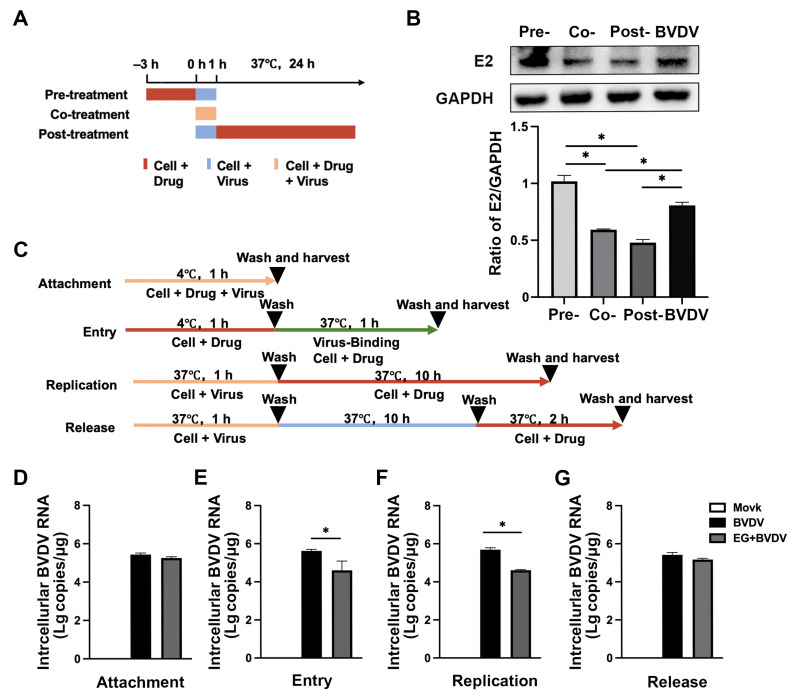
Effect of EG on inhibition of BVDV life cycle. (**A**) MDBK cells were treated with EG at 50 µM and BVDV (MOI = 1) infection schematic at time of addition assay. (**B**) The expression of BVDV E2 protein was collected from the cells at 24 hpi and analyzed by Western blot. (**C**) MDBK cells were treated with EG at 50 µM and BVDV (MOI = 1) infection schematic in the attachment, entry, replication, and release steps assays. (**D**–**G**) The BVDV 5′UTR mRNA level was analyzed by qRT-PCR. Data represent mean ± SEM (*n* = 3). * *p* < 0.05.

To further determine the role of EG in the BVDV life cycle, MDBK cells were incubated with EG at a concentration of 50 µM before (pre-treatment), at the same time (co-treatment), and after (post-treatment) the viral inoculation, respectively (Figure 3A). As shown by Western blot, compared to the control group, the co-treatment and post-treatment groups significantly reduced the expression of E2 protein, with no significant difference in the pre-treatment group (Figure 3B). In general, the viral life cycle is divided into four steps: adhesion, entry, replication, and release. Therefore, EG at a concentration of 50 µM was added to BVDV-infected MDBK cells at different time points, and the expression of BVDV 5′UTR mRNA was measured by qRT-PCR to further investigate how EG affects the stage of the BVDV life cycle (Figure 3C). According to our results, EG treatment had no effect on the attachment of the virus to cells, and the same results for the release phase (Figure 3D). But treatment with EG at the entry and replication stage significantly reduced BVDV 5′UTR mRNA levels (Figure 3D), suggesting that EG can impair BVDV during the entry and replication phases. The results described above showed that EG inhibits the early stages of BVDV infection by acting on the entry and replication of the viral life cycle.

### 2.3. EG Promotes the Production of IFITM3 in MDBK Cells Infected with BVDV

**Figure 4:**
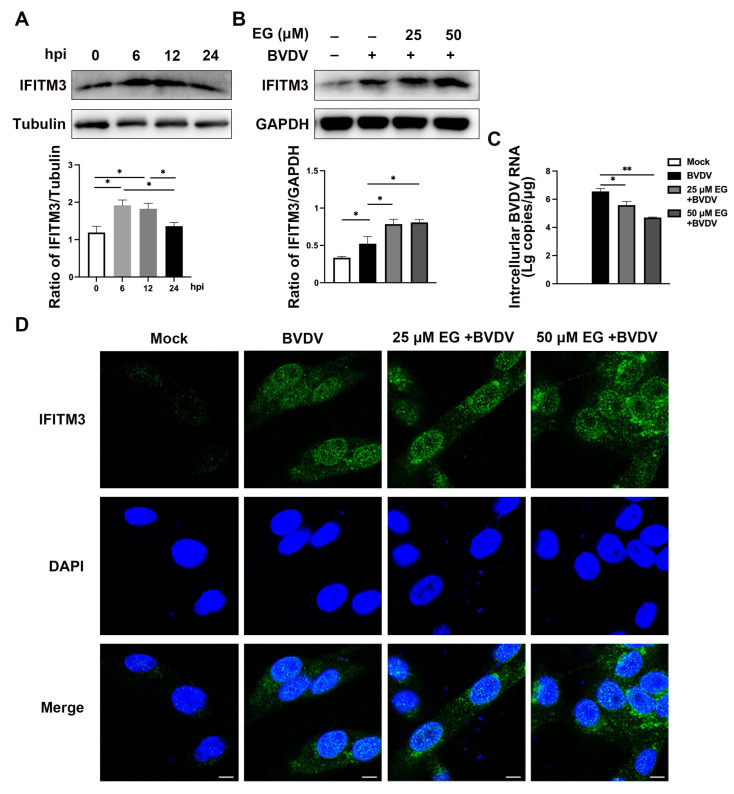
EG promotes the production of IFITM3 in MDBK cells infected with BVDV. (**A**) Western blot analysis of the protein expression of IFITM3 in MDBK cells infected with BVDV (MOI = 1) for 6 to 24 h. (**B**) MDBK cells were treated with EG at 25, 50 µM after BVDV infection, then incubated for 24 h. The protein expression of IFITM3 was detected by Western blot. (**C**) The BVDV 5′UTR mRNA level was analyzed by qRT-PCR. (**D**) The IFITM3 were analyzed by immunofluorescence assay with the antibody against anti-IFITM3 (green) and nuclear (blue). Scale bar, 50 µm. Data represent mean ± SEM (*n* = 3). * *p* < 0.05, ** *p* < 0.01.

IFITM3 is considered to have antiviral activity, especially against enveloped viruses, including HCV virus, influenza A virus, Ebola virus, dengue virus, and so on [21,22,23,24]. In previous studies, it was demonstrated that the IFITM expression level was upregulated after viral infection [25]. To understand IFITM3 protein expression after BVDV infection at multiple time points, we investigated the level of IFITM3 by Western blot. We found the IFITM3 protein level in MDBK increased at 6 hpi and peaked at 12 hpi, suggesting that the IFITM3 expression was upregulated before BVDV replication had occurred (Figure 4A). But the protein level of IFITM3 was reduced at 24 hpi. With this result, we speculate that upon sensing an attacking virus, host cells receive these signals, ultimately leading to the activation of an innate immune response, which mainly consists of the secretion of interferon (IFN) and the expression of antiviral factors [26]. To further evaluate whether EG acts as an anti-BVDV replication inhibitor by increasing IFITM3 expression, MDBK cells were co-treated with EG for 24 h, followed by BVDV infection. The addition of EG caused an increase in the expression of IFITM3 in MDBK cells, as shown in Figure 4B. The level of BVDV 5′UTR mRNA expression in EG-treated cells was significantly reduced compared to BVDV-infected groups (Figure 4C). Furthermore, we investigated the expression and distribution of IFITM3 by immunofluorescence assay. Following EG treatment, IFITM3 protein expression was increased in MDBK cells at concentrations of 25 and 50 µM by immunofluorescence microscopy (Figure 4D). As shown in Figure 4D, IFITM3 was mainly focused on the intracellular compartment of the cytoplasm after BVDV infection and EG treatment. These above results suggest that EG can promote the innate response in MDBK cells, which may take an essential role in the host resisting the BVDV infection in the early stages.

### 2.4. EG Promotes Lysosomal Acidification and Protease Activity to Prevent BVDV Infection

**Figure 5:**
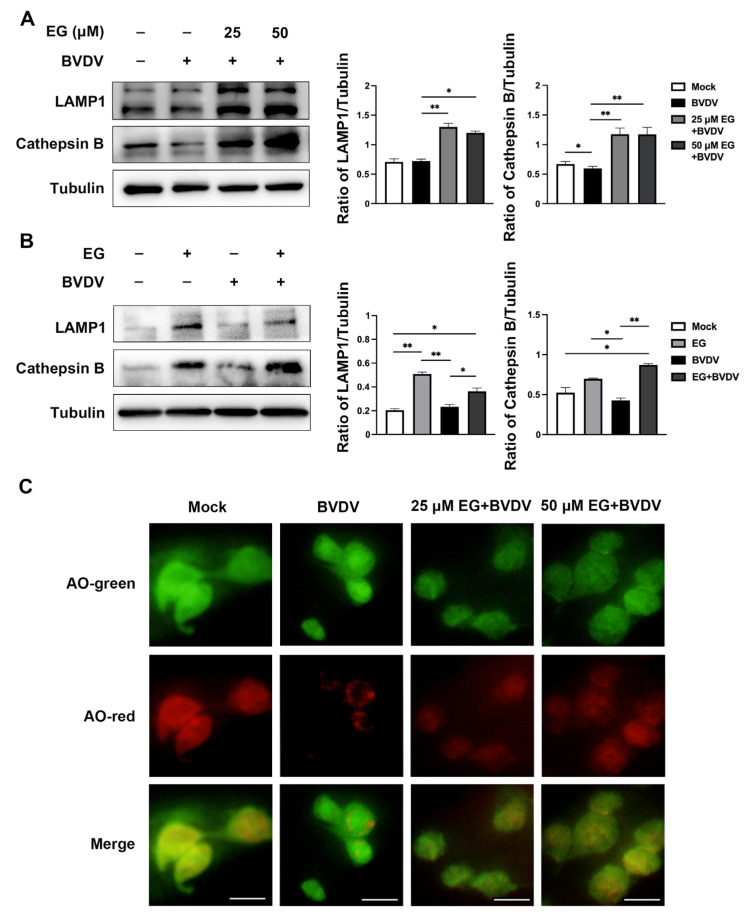
EG promotes lysosomal acidification and protease activity to prevent BVDV infection. (**A**) MDBK cells were treated with EG at 25, 50 µM after BVDV (MOI = 1) infection for 24 h. The protein levels of LAMP1 and cathepsin B were detected by Western blot, using Tubulin as a control. (**B**) MDBK cells were infected with BVDV and treated with EG at 50 µM for 24 h. The protein levels of LAMP1 and cathepsin B were assessed by Western blot, using Tubulin as a control. (**C**) MDBK cells were stained with acridine orange (AO) for 15 min and detected by immunofluorescence assay using a 488 nm (green) or a 561 nm (red) laser. Scale bar, 50 µm. Data represent mean ± SEM (*n* = 3). * *p* < 0.05, ** *p* < 0.01.

Studies have shown that IFITM3 prevents membrane fusion of viruses invading cells via the endocytic route [24]. IFITM3 binds to the vesicles of viral particles that have entered the cell and facilitates the late-stage endocytic cargo trafficking to the lysosome for degradation, which blocks viral invasion [13]. Therefore, we assessed whether EG accelerates the degradation of IFITM3-bound viral particle vesicles by enhancing lysosomal function. Previous reports have shown that overexpression of IFITM3 enhanced the staining of lysosomal-associated membrane protein 1 (LAMP1) and increased protein expression [8]. LAMP1, the late endosomal marker, exerts a positive effect on maintaining the structural integrity of the lysosome [27]. Cathepsin B is necessary for lysosome maturation. First, we evaluated the expression level of LAMP1 by Western blot and found that different concentrations of EG-treated MDBK cells significantly increased LAMP1 expression compared to the control and BVDV-infected group, as expected (Figure 5A,B). Then, BVDV infection significantly reduced the protein level of the maturated form of lysosomal hydrolase cathepsin B, as shown by Western blot (Figure 5A,B). The addition of EG after virus infection clearly increased the protein level of cathepsin B compared to the BVDV-infected group (Figure 5A,B). Here, we further examine the interaction of lysosomal acidification with BVDV or EG in MDBK cells by acridine orange (AO) staining, which exhibits red fluorescence when in the acidic lysosome. The fluorescence intensities of AO staining were significantly decreased in BVDV-infected cells but increased in EG-treated cells (Figure 5C), indicating that EG alleviates the disruption of lysosomal acidification by BVDV infection. These results indicated that EG might inhibit BVDV replication by promoting endo-lysosomal acidification and protease activity, thereby enhancing lysosomal degradation.

### 2.5. EG Limited Replication of BVDV by Interfering with Autophagy In Vitro

**Figure 6:**
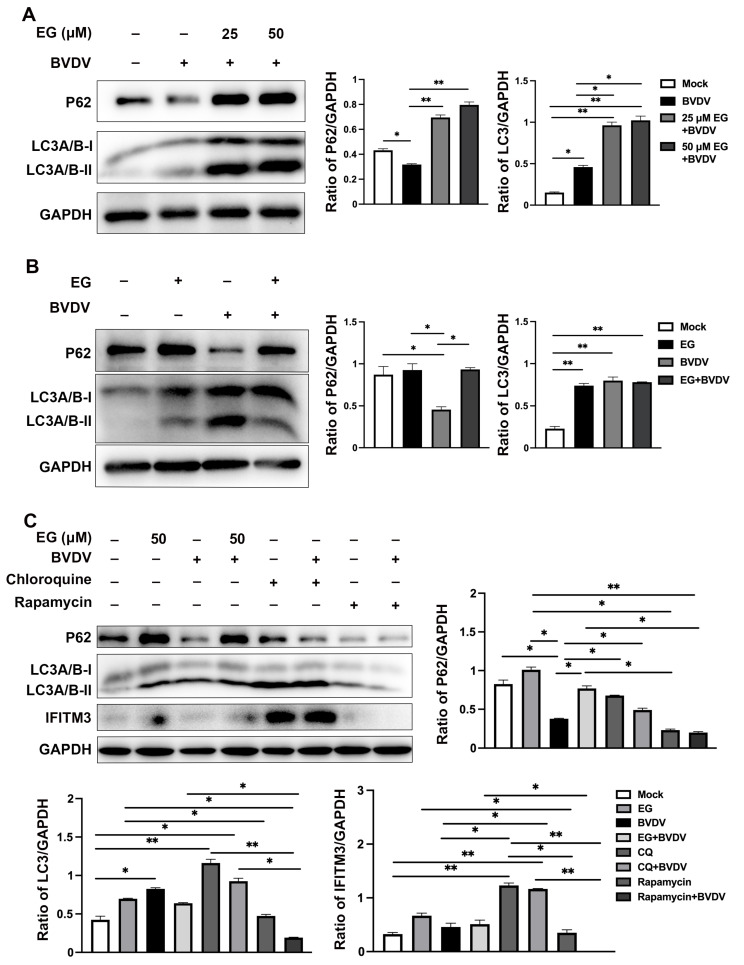
EG limits replication of BVDV by interfering with autophagy in vitro. (**A**) MDBK cells were infected with BVDV and treated with EG at 50 µM for 24 h. The levels of p62 and LC3 were detected by Western blot, using GAPDH as a control. (**B**) MDBK cells were treated with EG at 25, 50 µM after BVDV infection for 24 h. The levels of p62 and LC3 were detected by Western blot, using GAPDH as a control. (**C**) The expression levels of p62 and LC3 were detected by Western blot in MDBK cells treated with EG, CQ, Rapa, respectively. Data represent mean ± SEM (*n* = 3). * *p* < 0.05, ** *p* < 0.01.

A primary characteristic of autophagy is the stimulation of innate and adaptive immune responses to eliminate and destroy the invading virus [14,28]. Autophagy activates the innate immune response of the body by binding to pattern recognition receptors (PAMPs) signals to induce interferon production [14]. But autophagy can be a double-edged sword for viral infection, such as is the case for some RNA viruses that can use autophagy to complete their replication. In our previous work, we found that BVDV infection could activate the autophagic process, which facilitates viral replication [29]. Hence, we postulated that EG could potentially exhibit antiviral effects by modulating autophagy. The effect of EG on autophagy was detected in MDBK cells, which were treated with different concentrations of EG (25 and 50 µM), and Western blot analysis was applied to measure the protein expression levels of p62 and LC3. In comparison to the control group, the level of LC3 expression was upregulated in BVDV-infected or EG-treated groups (Figure 6A,B), indicating the accumulation of autophagosomes after BVDV or EG treatment. LC3 is essential for autophagosome formation and is used as an autophagosome marker. Then, we also measured the levels of p62 and found a significantly higher expression in EG-treated group compared to the control group, yet a significantly lower expression in the BVDV-infected group (Figure 6A,B). EG increased the levels of LC3 and p62, indicating that EG may block autophagic degradation. To support the evidence that EG may prevent that degradation caused by BVDV infection in MDBK cells, Chloroquine (CQ) and Rapamycin (Rapa) were used in the subsequent study. Rapa, an inhibitor of mTOR, activates autophagy [30]. CQ is a critical inhibitor of the late stage of autophagy, which can block autophagy by decreasing autophagosomal lysosomal fusion [31]. Western blot analysis suggested that CQ treatment significantly enhanced the expression levels of LC3 and p62, which was consistent with the EG treatment group results (Figure 6C). Interestingly, LC3 protein expression was enhanced, and p62 protein expression was decreased, in the Rapa-treated group compared to the control group, with the same result for the BVDV infection (Figure 6C). Of note, we examined the expression of IFITM3 in MDBK cells and observed a remarkable upregulation of IFITM3 following treatment with CQ or EG, and a significantly reduction in the level of IFITM3 following Rapa treatment (Figure 6C). This data suggests that EG might regulate IFITM3 expression through autophagy. According to the above results, we hypothesized that BVDV induces autophagic flux, whereas EG might block the autophagy in the late stage, indicating that EG inhibits viral replication by blocking autophagy induced by BVDV. However, more studies are required to explore the relationship between IFITM3 and autophagy.

## 3. Discussion

The pathogenesis of BVDV-infected cattle is complex, with persistent infection and immunosuppression, making it hard to eradicate BVDV and causing serious economic losses to the dairy cattle industry when infections arise [4]. Due to the immune evasion ability of BVDV, the need for research on antiviral drugs has become imperative. EG is a chemical monomer that exists in a variety of natural medicinal plants, which is an organic compound of polyhydroxyphenols. In earlier studies on the antiviral activity of gallate derivatives, some showed that EGCG can fight against a variety of viral infections [20,32,33]. However, the inhibitory action of EG on BVDV infection and the mechanisms of its role have not been completely clarified. In this study, we illustrate the anti-BVDV effect of EG and the mechanisms related to it.

Based on the BVDV growth curve, the number of viruses grows steadily within 12 hpi of BVDV inoculation, and from 12 to 24 hpi is the logarithmic growth stage [34]. These results suggested that EG could block BVDV infection from 6 to 24 hpi, indicating that EG inhibits the initial phase of the BVDV life cycle, as well as its logarithmic growth phase. Intriguingly, the antiviral activities of EG at a concentration 50 µM against BVDV in vitro was evidently stronger. To investigate the effect of EG on the BVDV life cycle, EG was added to BVDV-infected MDBK cells at different time points, and the expression of BVDV 5′UTR mRNA was measured. The results indicated that treatment with EG inhibits the entry and replication phases of the BVDV infection life cycle in MDBK cells. However, it remains unclear how EG prevents the infection of BVDV.

IFITM, which is abundant in animal cells, belongs to the CD225 protein superfamily and can be strongly induced during viral infection [35]. Some studies suggest that IFITM proteins block the infectious entry of multiple enveloped viruses by preventing viral fusion at the membranes of the plasma or endolysosomes [25,36]. To investigate whether BVDV influences IFITM3 expression, we decided to collect samples at 6, 12, and 24 h after BVDV infection of MDBK cells, and then detected the protein expression of IFITM by the Western blotting method. The results showed that BVDV infection activates IFITM3, and that IFITM protein expression increased at 6 hpi and peaked at 12 hpi, suggesting that MDBK cells upregulate IFITM3 expression before BVDV completes replication. We therefore further investigated whether EG could affect IFITM3 expression in MDBK cells while infected with BVDV. It is noteworthy that treatment of BVDV-infected MDBK cells with EG increased the protein expression level of IFITM3. We then analyzed the subcellular localization of IFITM3 protein. The data revealed that IFITM3 is primarily located in the cytoplasmic and circum-nuclear regions after being treated with EG or BVDV, which is consistent with observations for other flaviviruses. These data may explain the anti-BVDV effect of EG in the early stage by its increasing of the expression of IFITM3, which is induced by IFNs that indicate enhanced immune response. But the mechanism of how IFITM3 inhibits BVDV infection needs further investigation.

The main function of lysosomes is to degrade substances that enter the cell through endocytosis, phagocytosis, or autophagy pathways. IFITM3 protein is mainly located in the membranes of endocytic vesicles, plasma membranes, and lysosomes [37]. Research has shown that IFITM3 proteins inhibit the propagation of CSFV and are associated with lysosomes [9]. Thus, the role of IFITM3 in suppressing viral infection is thought to be critically linked to the lysosome, and lysosome acidification and protease activity have been identified in association with some viral infections [38,39]. IFITM3 overexpression or IFN stimulation leads to increased lysosomal amplification and viral inhibition [8]. These suggest that IFITM3 exhibits antiviral activity via endosomal pathways. In the present study, BVDV blocked lysosomal compartment acidification and protease activity, followed by a promotion of BVDV replication. Therefore, we speculate that IFITM3 inhibits BVDV infection by disrupting the BVDV late endosomal pathway [9]. Furthermore, we investigated the impact of EG on lysosomes. The lysosome membrane glycoprotein LAPM1 expression was detected by Western blot, indicating that EG could maintain the membrane structural integrity of lysosomes. Then, we investigated the effect of EG on protease activity and lysosomal acidification, using Western blot to detect the protein level of cathepsin B and AO staining to detect lysosomal acidification, respectively. The results showed that the cathepsin B expression in the EG-treated group was significantly increased compared with that in BVDV-infected group. The fluorescence intensities of red revealed that treatment with EG increased the lysosomal acidification when compared with BVDV-infected group. These observations might be explained by the fact that EG efficiently inhibits BVDV replication in MDBK cells by maintaining the acidification and degradation capacity of lysosomes.

The autophagy pathway is a highly dynamic pathway and is induced when cells are exposed to stressful conditions such as hypoxia, starvation, and infection by pathogenic microorganisms. However, much evidence indicates that certain viruses may utilize the autophagic pathway to achieve their own proliferation, such as dengue virus, HCV, and BVDV [28,29]. Our data showed that NCP-BVDV could induce autophagy formation, which was detected by the increase of microtubule-binding protein light chain 3 (LC3) expression, consistent with other previous studies [1,29,40]. This suggests that BVDV may utilize autophagy to facilitate viral reproduction. Thus, we further detected whether EG blocks BVDV infection by affecting autophagy. The data suggest that EG treatment can significantly upregulate the protein expression levels of LC3 and P62 compared with the control or BVDV-infected group, demonstrating that EG may inhibit BVDV infection by regulating autophagy flux in MDBK cells. LC3 as a marker of autophagosome formation. P62, bound with LC3-II, is a particular cargo protein of the autophagosome for forming aggregates prior to transport. Subsequently, to further confirm the role of EG in preventing BVDV infection by autophagy, autophagy activator Rapa and inhibitor CQ were treated with MDBK cells, respectively. Rapa, an mTOR inhibitor, can promote autophagic flux. mTOR inhibitors extensively degrade cell-associated proteins through activation of the proteasome and autophagic pathways [41]. CQ, a weak base, is widely used to inhibit the later stage of autophagy. Our previous study found that Rapa promotes autophagy to upregulate BVDV E2 expression, whereas CQ inhibits autophagy to downregulate [29]. In this study, MDBK cells treated with EG significantly enhanced protein expression levels of LC3 and P62, with the same results for CQ. This showed that EG inhibits BVDV infection by blocking the late stage of autophagy. Studies have found that the overexpression of IFITM3 leads to elevated autophagosome marker LC3-II [42]. Then, we tested the effects of both Rapa and CQ on IFITM3 protein expression, indicating that CQ significantly enhanced IFITM3 expression while Rapa significantly degraded it. This data suggests that EG might regulate IFITM3 expression through autophagy. However, further experiments in more detail are needed to understand the relationship between IFITM3 and autophagy.

## 4. Materials and Methods

### 4.1. Reagent and Antibodies

Chloroquine (CQ) was purchased from Sigma-Aldrich (St. Louis, MI, USA). Rapamycin (Rapa) was purchased from MedChemExpress, (South Brunswick, NJ, USA). Acridine orange (AO) was purchased from Solarbio (Beijing, China). EG was purchased from Macklin (Shanghai, China).

The antibodies used in this study were anti-Fragilis (Huabio, Hangzhou, China), anti-LC3A/B (Cell Signaling Technology, Danvers, MA, USA), anti-cathepsin B polyclonal antibody (Wanleibio, Shenyang, China), anti-BVDV E2-specific mouse monoclonal antibody (VMRD, Pullman, WA, USA). Additionally, anti-LAMP1 (21997-1-AP), anti-SQSTM1/p62 (18420-1-AP), anti-GAPDH (60004-1-AP), and anti-Tubulin (10068-1-AP) were purchased from ProteinTech Group Inc. (Rosemont, IL, USA). Secondary antibodies in this study included Alexa Fluor 488-Conjugated Goat Anti-Rabbit IgG (Beyotime, Shanghai, China). Horseradish peroxidase (HRP)-conjugated goat anti-mouse IgG (SA00001-1), and anti-rabbit IgG (SA00001-2) were purchased from ProteinTech Group Inc. (Rosemont, IL, USA).

### 4.2. Cell Culture and Viruses

Madin-Darby Bovine Kidney (MDBK) cells were cultured in Dulbecco’s Modified Eagle Medium/Ham’s F-12 Medium (DMEM/F-12) (Gibco, Grand Island, NY, USA) complemented with 5% fetal bovine serum (FBS) (Gibco, Fitzroy North, Victoria, Australia) and 1% antibiotic-antimycotic (Gibco, Grand Island, NY, USA), at 37 °C in 5% CO_2_. The NCP-BVDV-BJ175170 strain was previously isolated and preserved in our laboratory. MDBK cells were infected with the NCP-BVDV-BJ175170 strain at an MOI of 1. Unbounded viruses were eliminated by washing with PBS after 1 h incubation at 37 °C, and then maintained in DMEM/F-12 complemented with 2% FBS at 37 °C for 72 h. After three cycles of freezing and thawing, the viral cells were centrifuged at 12,000× *g* for 15 min at 4 °C.

### 4.3. The Effect of EG Treatment at Different Times

To investigate the effectiveness of EG in inhibiting BVDV infection, MDBK cells were incubated with EG (50 µM) at various stages of viral infection (MOI = 1).

Pre-treatment group: MDBK cells were pretreated for 3 h with EG and then washed with PBS three times before being infected with BVDV for 1 h at 37 °C under 5% CO_2_. The cells were cultured in a maintenance medium (DMEM/F-12, 2% FBS, 1% antibiotic-antimycotic) for 24 h.

Co-treatment group: The cells were incubated with EG and BVDV concurrently for 1 h and then cultured in maintenance medium for 24 h at 37 °C.

Post-treatment group: To investigate whether EG had any effects after viral entry, cells were incubated with BVDV for 1 h. Afterward, infected cells were washed with PBS and subsequently overlaid with maintenance medium containing EG for 24 h at 37 °C.

### 4.4. Virus Replication Cycle Assay

Attachment assay: An attachment assay was performed to evaluate the effect of EG on BVDV adsorption. The cells were co-treated with EG or DMSO and BVDV at 4 °C for 1 h, which enabled the virus to adhere to cells but prevented invasion. The mRNA levels of BVDV 5′UTR were analyzed by qRT-PCR.

Entry assay: The cells were infected with BVDV and incubated at 4 °C for 1 h to allow virus binding. Then, treated cells were washed with PBS three times to remove unbound viruses before treatment with EG or DMSO for 1 h at 37 °C. The PBS (pH = 3.0) was used to wash MDBK cells to remove drugs and non-internalized extracellular viruses. The mRNA levels of BVDV 5′UTR were analyzed by qRT-PCR.

Replication assay: To analyze drug effects post viral entry, cells were incubated with BVDV at 37 °C for 1 h. Extracellular viruses were removed by washing the treated cells with PBS (pH = 3.0) three times. The cells were incubated at 37 °C for 10 h in DMEM/F-12 complemented with 2% FBS and EG. The mRNA levels of BVDV 5′UTR were analyzed by qRT-PCR.

Release assay: MDBK cells were incubated for 1 h with BVDV at 37 °C. Then extracellular viruses were removed by washing the treated cells with PBS (pH = 3.0) three times. The wells were then covered with maintenance medium at 37 °C for 10 h. The cells were washed with PBS three times and in the maintenance medium was added EG. The treated cells were incubated at 37 °C for 2 h. The progeny viruses in culture medium supernatant were collected and we analyzed the mRNA levels of BVDV 5′UTR by qRT-PCR.

### 4.5. Cytotoxicity Assay

Cell viability was evaluated using Cell Counting Kit-8 (CCK-8) assay (Beyotime, Beijing, China). MDBK cells were cultured in 96-well plates at a 2 × 10^4^ cells/well for 24 h at 37 °C under 5% CO_2_. Then the medium was changed to DMEM/F-12 complemented with 5% FBS, 1% antibiotic-antimycotic, and EG (0–100 µM) for 24, 48, and 72 h. Lastly, to each well was added 10 μL CCK-8 and they were incubated for 3 h at 37 °C according to the manufacturer’s instruction. The absorbance of 450 nm was detected using a microplate reader. All tests were performed three times.

### 4.6. Quantitative Reverse Transcription-PCR (qRT-PCR)

Total RNA was isolated from MDBK cells using the Omega total RNA kit (R6834, Guangzhou, China). For the cDNA synthesis, 1 µg of total RNA was used for reverse transcription by using a TransStart one-step gDNA removal and cDNA synthesis supermix kit (AT311-02, TransGen Biotech, Beijing, China). Quantitative real-time RT-PCR was performed using the 10 µL SYBR Green PCR Master Mix (LS2062, Promega, Madison, WI, USA), 0.5 µL of forward or reverse primer, 1 µg cDNA, and an addition of H2O to a final reaction volume of 20 µL. This study used the following primers: Glyceraldehyde-3-phosphate dehydrogenase (GAPDH) (forward: 5′-AAAGTGGACATCGTCGCCAT-3′, reverse: 5′-CCGTTCTCTGCCTTGACTGT-3′), BVDV 5′UTR (forward: 5′-TAGTCGTCAGTGGTTCGACGCC-3′, reverse: 5′-CCTCTGCAGCACCCTATCAG-3′). IFITM3 (forward: 5′-ATCCCAGCCCTTGTTCACTG-3′, reverse: 5′-GACACGAGGACGATGAGGAC-3′). The results were analyzed by the cycle threshold (CT) method.

### 4.7. Western Blotting

Radioimmunoprecipitation assay (RIPA) buffer (Solarbio, Beijing, China) that contains protease/phosphatase inhibitor cocktail (Cell Signaling Technology, Danvers, MA, USA) was used to lyse cells at 4 °C for 20 min. Using the Pierce BCA protein assay kit (ThermoFisher Scientific, Waltham, MA, USA), protein concentration was measured. Total proteins were separated by sodium dodecyl sulfate polyacrylamide gel electrophoresis (SDS-PAGE) and then transferred to polyvinylidene fluoride (PVDF) membranes, pore size 0.45 µm (Millipore, Bedford, MA, USA). Membranes were blocked with 5% (*w*/*v*) skimmed milk powder in PBS containing 0.05% Tween 20, then incubated with the primary antibody overnight at 4 °C. The membranes were incubated with the secondary antibody for 1 h at room temperature. Immunoreactive bands were visualized using an ECL detection system (Tanon Technology Co., Ltd., Shanghai, China).

### 4.8. Immunofluorescence Assay (IFA)

MDBK cells were seeded in 24-well plates on glass coverslips and treated with 4% paraformaldehyde for 20 min at room temperature. After washing three times, cells were permeabilized with 1% (*v*/*v*) Triton X-100 (T8787, Sigma-Aldrich, St. Louis, MI, USA) for 15 min, and blocked in PBS with 2% (*w*/*v*) bovine serum albumin for 1 h. The cells were incubated with primary antibodies at 4 °C overnight. Then, the cells were incubated with Alexa Fluor 488 goat anti-rabbit secondary antibodies (Beyotime Biotechnology, Shanghai, China) for 1 h at room temperature. 4′-6-diamidino-2-phenylindole (DAPI, C0060, Solarbio Science & Technology Co., Ltd., Beijing, China) was used to stain nuclei. Images were acquired by confocal microscopy.

### 4.9. Acridine Orange Staining

MDBK cells were arranged flat on glass coverslips in 24-well plates for 24 h prior to treatment. Then, treated cells were dyed using AO solution (5 µg/mL) for 20 min at 37 °C in the dark. We washed the dyed cells three times with PBS and covered them with sheets after dyeing. The fluorescent signal was observed by confocal microscopy at 488 nm (green) or 561 nm (red).

### 4.10. Statistical Analyses

We used GraphPad Prism 9 software for the statistical analysis. Data were presented as the mean ± standard deviations (SD). Statistical significance was analyzed by one-way analysis of variance (ANOVA) or Student’s *t* test.

*p* < 0.05 was regarded as significant. Data were repeated independently at least three times.

## 5. Conclusions

In conclusion, the above results show that EG at a concentration of 50 µM could potently inhibit the BVDV infection of MDBK cells. Furthermore, EG has been shown to inhibit viral infection by suppressing the entry and replicative phases of the BVDV life cycle. The mechanism of EG against viral infection works by increasing IFITM3 expression, lysosomal protease activity, and lysosome acidification. Meanwhile, EG was found to suppress autophagy of NCP-BVDV induced in MDBK cells. Our result provides a rational explanation for the possibility of utilizing natural product extracts to develop new strategies against BVDV infection.

## Data Availability

Not applicable.
